# AI-driven skill volatility and the emergence of re-skilling fatigue: the human cost of perpetual learning

**DOI:** 10.3389/frai.2026.1785979

**Published:** 2026-05-08

**Authors:** Tittu Elizabath Biju, Anusree Ambady, Thomas K V

**Affiliations:** 1 Department of Commerce, Marian College Kuttikkanam Autonomous, Kuttikkanam, India; 2 Department of Commerce, BCM College Kottayam, Kottayam, India

**Keywords:** artificial intelligence, conservation of resources theory, established knowledge professionals, job demand-resources theory, reskilling fatigue

## Abstract

Artificial intelligence (AI) is rapidly reshaping knowledge-intensive work by automating, augmenting, and reconfiguring core professional activities. While continuous reskilling is widely promoted as a solution to AI-driven disruption, little attention has been paid to its cumulative psychological costs. This paper introduces the concept of reskilling fatigue to explain the human consequences of persistent skill volatility among Established Knowledge Professionals (EKPs), mid-career professionals whose roles, identities, and value are grounded in accumulated expertise and professional judgment. Based on Job Demands–Resources (JD-R) theory and Conservation of Resources (COR) theory, the paper conceptualizes an AI-induced reskilling loop in which ongoing technological change leads to skill erosion, continuous reskilling demands, cognitive and emotional depletion, and reinforced learning as a defensive response to perceived obsolescence. Unlike restoring stability, this cycle intensifies anxiety, undermines mastery, and erodes professional confidence. As a contribution to theory and practice, the paper advances a set of sustainable, collective strategies such as role-linked learning, protected learning time, skill prioritization, and phased AI adoption to interrupt the reskilling loop and redistribute adaptive demands across organizations. By reframing reskilling as a shared, supported, and bounded process, this paper highlights pathways through which AI-driven change can foster long-term career resilience, professional identity renewal, and sustainable human–AI integration.

## 1 Introduction

The rise of artificial intelligence will be rewriting the way we work through automation, through changes in how we do our jobs and as a result, through changes in how we will experience our jobs, and the speed at which we develop the skills necessary for success. Increasingly, as artificial intelligence takes over the roles of decision making, analytical thinking, and coordination of human beings, the experience gained from education and work experience will lose its value. As a result, reskilling will be the primary avenue for professional survival and virtually a mandatory action taken by both organizations and policymakers. With a focus on continuous learning, the obligation to engage in learning and development will be greatest for those workers who have built their professional identity and competence from years of accumulated knowledge and experience (Sharma et al., 2025). The discourse around reskilling typically focuses on the empowerment a worker receives through this process, but little research exists on the psychological impact of reskilling fatigue, which exists in the information technology and workforce literature. These studies significantly focus on the positive effects of reskilling but ignore the cognitive, emotional and time-consuming requirements cumulated over time due to the demands of reskilling, causing fatigue to workers. This paper posits that reskilling fatigue is not a personal, but rather a systemic result of the design of AI-enabled work. Continuous reskilling creates a constant job demand that drains human resources and drives job insecurity and creates an environment for continued anxiety and fatigue. Following the Job Demands-Resources (JD-R) model and the theory of Conservation of Resources (COR), the proposed model of reskilling fatigue will reframe the process of reskilling as a finite human resource.

## 2 Manuscript formatting

### 2.1 Methodological approach

Aligning with the objectives of perspective article, this study adopts a theory-driven conceptual methodology. Instead of empirical data, analysis of this study combines and interprets various interdisciplinary literature on artificial intelligence, work transformation, occupational stress, and workforce sustainability. The integration of application of JD-R model and COR theory to contemporary AI-incorporated work settings, supports the argument, by specifically focusing Established Knowledge Professionals (EKP). Relevant peer-reviewed studies based on occupational psychology, human resource management and AI-in-society research were particularly examined to understand the continuous patterns of skill volatility, reskilling expectations and psychological strain. This paper uses an interpretive synthesis approach to conceptualize AI-induced reskilling fatigue loop as a heuristic design to explain how the interplay of increased learning demands and threatened resources leads to sustained anxiety and exhaustion.

### 2.2 Theoretical framework

JD-R model can be applied to understand how work characteristics influence psychological strain and well-being (Demerouti et al., 2001). Hence, the same can explain the emergence of reskilling fatigue among EKPs. As per the JD-R framework, job demands include work aspects that need prolonged physical or mental efforts and so are connected with psychological costs, whereas job resources include aspects that aid in achieving work goals, reduce demands and foster growth and engagement. When demands consistently dominates resources, employees feel strain, exhaustion and disengagement which leads to burnout (Bakker and Demerouti, 2017). In the context of AI-driven work, persistent reskilling becomes a chronic job demand which requires continuous mental effort, time and emotional energy to fulfill professional duties. As a result, learning has become more of a compulsory performance requirement rather than a resource-enhancing activity. This aligns with the extensions of the JD-R theory that highlight how enduring high demands exceeding resources lead to health problems and reduced psychological well-being (Bakker et al., 2005).

Further, COR theory helps in understanding how reskilling fatigue develops as a psychological response to the continuous and expected resource loss due to AI-driven work exposure. As per COR theory, individuals seek to acquire and safeguard their precious personal and work-related resources. When these resources are threatened, depleted or failed to restore despite ongoing efforts, stress occurs in the, minds of individuals (Halbesleben et al., 2014). Considering these circumstances, the risk of obsolescence and job redundancy warns a potential loss of resources, which prompts the professionals to undertake reskilling as a protective strategy. However, newly learned skills quickly become outdated and learning efforts fail to produce lasting benefits which makes continuous resource investment without sufficient return. This disequilibrium creates emotional exhaustion among individuals and reduces their motivation. Together, JD-R and COR theories suggest that increasing job demands through continuous reskilling, combined with shrinking or unstable resources, create a loss spiral in which strain intensifies over time, ultimately manifesting as reskilling fatigue as a systemic condition rather than an individual shortcoming (Demerouti, 2025).

Reskilling fatigue has some similarities to constructs such as burnout and technostress, but it is a qualitatively different phenomenon that has arisen in artificial intelligence-driven workplaces. While burnout is caused by excessive job demands (e.g., workload, emotional exhaustion, long hours of work) (Gulalp et al., 2008), technostress is caused when employees have difficulty adjusting to new technology (Bahamondes-Rosado et al., 2023; Wang and Yao, 2025). In contrast, reskilling fatigue results from the continual instability of knowledge and expertise due to rapid technological changes. In AI-enabled workplaces, professionals must continuously update their competencies because previously learned skills are constantly becoming obsolete at an accelerating rate. As a result of this ongoing process of reskilling, learning becomes a continuous job demand for employees, competing with employees’ current job responsibilities and placing additional strain on employees’ cognitive resources, under JD-R perspective. In addition, COR theory suggests when individuals repeatedly invest time and effort into reskilling without receiving any tangible, long term returns due to new skills becoming obsolete, this will create a resource loss spiral which develops into reskilling fatigue (Nastjuk et al., 2024). Thus, the strain associated with reskilling fatigue is not due to employees performing work tasks or interacting with technology, rather it is due to the unstable nature of skills within AI-driven labor market.

### 2.3 Established knowledge professionals and reskilling fatigue in AI-driven work

EKPs are mid-career individuals with between 10 and 20 years of experience in their respective professions. They fill the gap between the early career professionals, whose major preoccupations are skill formation and late career professionals who are in the consolidation or departure stages (Kim et al., 2024). They are characterized by deep domain expertise, role mastery and relatively stable professional identities which are grounded in accumulated knowledge and judgment. However, radical transformations enabled by AI through automation, augmentation, and even the redefining of main work activities weaken the link between established expertise and employability. Thus, the need for adaptation has shifted from an optional decision to a structural demand for EKPs, as upskilling and reskilling must be continuous to avoid career stagnation. This creates insecurity or anxiety among them, which is structural, not episodic, arising from persistent concerns of obsolescence in skills, displacement in tasks, and erosion of long-established professional competence (Salari et al., 2025; Sharma et al., 2025). This anxiety is both anticipatory and experiential, not only due to expectations of ongoing technological changes but also to uncertainties about returns accruing from earlier investments in human capital. Empirical evidences support that such conditions deteriorate job satisfaction, organizational commitment and psychological wellbeing, resulting emotional exhaustion, pessimism and withdrawal intentions among employees operating in AI-intensive environments (Chen et al., 2025; Salari et al., 2025).

In the context of this framework, ongoing reskilling is identified as a major method for coping with employability, however it also results in what is referred to as reskilling fatigue, which is a cumulative depletion of cognitive, emotional, and motivational resources that results from continual anxiety-induced demands to learn, and with no opportunity to further consolidate the knowledge obtained. Fear about AI often push people to learn new skills, initially encouraging them to reskill to stay relevant, but eventually this perpetual learning drains out the durable sense of mastery, leading to increased stress (Kim et al., 2025). EKPs are particularly susceptible to reskilling fatigue because their job value is based on maintaining a stable level of expertise, however, frequent losses and gains of skill create financial and mental costs associated with both workforce readiness and psychological health as a result of the changing nature of AI-centered workplaces.

### 2.4 AI-induced reskilling loop among established knowledge professionals

EKPs observe a perpetually changing cycle within their professional roles as a result of the integration of artificial intelligence into their working environments. The cycle starts with an impetus for change that is initiated by AI fuelled by development and continues with the adoption of AI into organizations framework which ultimately begins to replace its core functions and restructure them. While such changes can be small and take place over time, they drastically alter how critical decisions are made as well as what constitutes performance and even redefine which expertise is most relevant. This type of change seeks to redefine their roles and thus calls for a reassessment of the boundaries and expectations set through experience, knowledge, and judgment on a professional basis. The effects of this disruption are ironically not an outright loss of jobs in the short run. Rather, they may begin to notice a gradual erosion of the skills and expertise they had built over time. To overcome this challenge, they might be forced to hastily learn new technologies, platforms, and even analytical approaches that would suit their changed roles. The process of skill displacement requires reskilling and ironically has made itself a natural component of professional existence. Moreover, such continuous reskilling has to be conducted along with existing professional roles, managerial tasks and financial obligations that are highly time-consuming, placing significant strain on the attention and cognitive capacity of EKPs. Hence reskilling is construed as an ongoing battle against becoming obsolete professionally.

**Figure 1:**
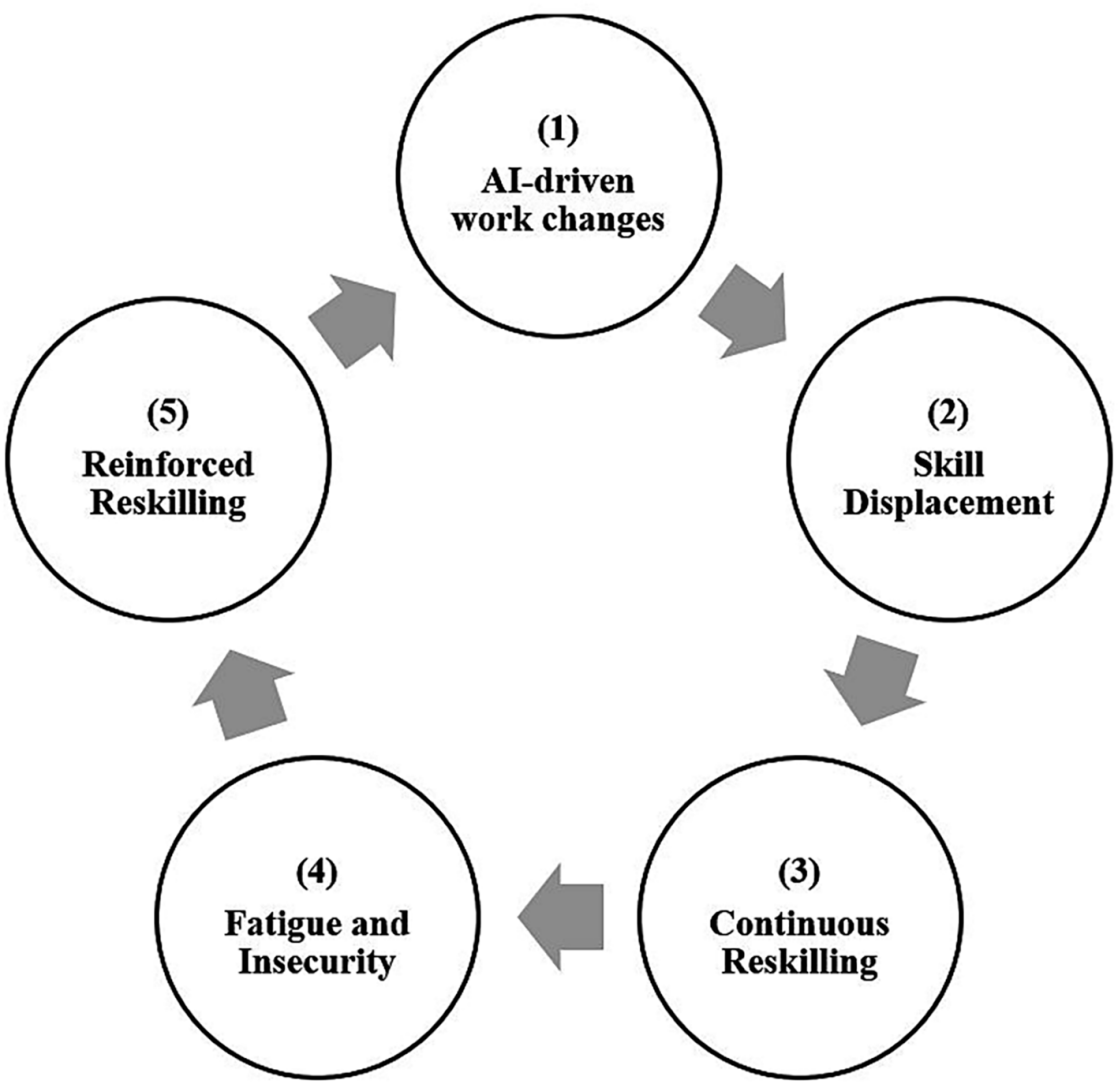
Reskilling loop. Source: Author compilations.

Furthermore, reskilling without the chance to consolidate skills can lead to fatigue and insecurity among EKPs. When the newly acquired skills are outpaced by rapid changes in technology, the process of reskilling creates nothing but a lack of a sense of competence and reinforces feelings of fragility. In these situations, reskilling becomes more of a strain rather than a positive growth experience. In order to alleviate this strain, they engage in reinforced reskilling, by increasing the number of learning activities. Here, learning has become more defensive against a looming sense of obsolescence. It has also become a means of self-preservation, as they attempt to protect themselves from the anxiety of being left behind. However, when organizations continue to alter functions to incorporate new technologies, this may lead to a repetitive cycle that is difficult to break. Hence, the outcome is a closed loop (Figure 1) of AI-driven workplace changes—skill displacement—the need for continuous reskilling—leading to fatigue and insecurity—renewed reinforced reskilling, in which change is habitual and a true sense of professional stability is impossible to achieve.

The reskilling loop becomes pathological once there is not enough stable expertise from learning but rather there is only a continuous replacement of skills. In AI-centric work contexts, new competencies devalue before there is sufficient time to build them into professional expertise. Therefore, most professionals continuously learn because they feel their skills will become obsolete rather than because they wish to receive regular personal growth. As time progresses, professionals create a self-reinforcing cycle where the continuous reskilling of their time and cognition will yield less and less value until they are both exhausted and insecure.

## 3 Discussion

Breaking the reskilling loop requires shifting the perspective on reskilling from an individual act to a collective, systemic, and sustainable process (Bankins et al., 2024). The challenges faced by EKPs are not primarily due to resistance to learning but rather arise from structurally reinforced patterns of AI-driven workplace change. Emerging empirical evidence further suggests that the rapid diffusion of artificial intelligence technologies is accelerating skill obsolescence and increasing pressure on professionals to update their competencies while managing existing work demands continuously (Chen et al., 2025; Salari et al., 2025). The following discussion (Table 1) outlines practical strategies for redesigning reskilling as a shared and supportive organizational practice.

**Table 1:** Sustainable strategies for breaking the reskilling loop.

Sustainable strategy	Practice	Implementation mechanism	Sustainable impact
Reskilling boundaries	Defining when reskilling is necessary and when it is not	Conduct biannual skill audits and schedule reskilling cycles every 6–12 months aligned with major AI system updates	Avoids endless, reactive learning cycles
Protected learning time	Designating learning hours allocated only for learning	Allocate 5–10% of weekly work hours (2–4 h/week) for structured learning integrated into official work schedules	Mitigates the struggle between learning and work demands
Skill prioritization	Limiting the focus to a defined set of critical, transferable skills	Limit training to 3–5 priority AI-related competencies per role per year based on organizational skill frameworks	Reduce cognitive overload and restore order
Role-linked learning	Linking reskilling to clearly defined role evolution	Introduce role-based learning pathways updated annually and linked to formal career progression reviews	Move from learning for survival to learning for growth
Valuing expertise	Recognizing and valuing experience in mentoring, expert tracks, or hybrid roles	Allocate 10–15% workload time for mentoring and knowledge-sharing roles for senior professionals	Protects professional identity and confidence
Phased AI adoption	Phased introduction of AI tools instead of constant upgrades	Introduce no more than 1–2 major AI tools per quarter, with 3–6-month stabilization periods between deployments	Allows consolidation and recovery from learning demands
Peer learning communities	Shared learning spaces and collective sense-making	Establish monthly peer learning sessions or internal AI forums where employees share tools, practices, and solutions	Reduces isolation and emotional strain

Source: Author compilations.

These remedies reduce urgency, restore a sense of predictability, and divide the adaptive burden, which helps in breaking the reskilling loop. However, they do not aim to eliminate reskilling, but to transform this process into a systematic supported effort that EKPs can sustain over time. Importantly, addressing fatigue due to reskilling requires an understanding that the continual need to adapt to ever-changing technology is not just an individual responsibility, it is also influenced by the organization’s decisions about how fast to adopt and implement AI technologies. This highlights the need for greater organizational accountability and governance in the design and implementation of AI-enabled workplace environments with structured, shared, and realistically supported learning demands. Moreover, when reskilling becomes slow-paced, purposive, and matches the changing nature of roles, it regains its developmental value and brings renewed confidence rather than exhaustion. Hence, it serves as an agent of professional growth, identity renewal, and long-term career resilience, allowing them to work positively with AI-driven changes, rather than viewing it as a persistent threat.

## 4 Scope for further research

To further develop this research pathway, future studies should look at how the AI-driven reskilling fatigue loop operates within various environments, industries and career stages so as to test their generalizability and boundaries. Longitudinal and mixed-method empirical research on how continuous reskilling requirements evolve over time and how they interact with various factors including the design of work, the nature of employee support from the organization, and the individual employees’ capacity for reskilling would also be valuable. There is a need for further research into how best to define and measure reskilling fatigue as an independent construct, distinguishing it from burnout and technostress as well as understanding its implications for employees’ levels of engagement, retention and career sustainability. As such, additional intervention-based research will seek to explore the potential of developing AI-supported human-centered approaches to design the learning environments in which employees are being reskilled, the use of boundary taxonomy as a tool for creating reskilling plans or reskilling portfolios and the role of organizational policies in relation to supporting employees to reskill in AI-enabled workplaces while also providing them with the capacity to adapt to future job demands.

## Data Availability

The original contributions presented in the study are included in the article/supplementary material, further inquiries can be directed to the corresponding authors.
